# Learned predictiveness training modulates biases towards using boundary or landmark cues during navigation

**DOI:** 10.1080/17470218.2014.977925

**Published:** 2014-11-20

**Authors:** Matthew G. Buckley, Alastair D. Smith, Mark Haselgrove

**Affiliations:** School of Psychology, University of Nottingham, Nottingham, UK

**Keywords:** Spatial navigation, Learning, Attention, Geometric module

## Abstract

A number of navigational theories state that learning about landmark information should not interfere with learning about shape information provided by the boundary walls of an environment. A common test of such theories has been to assess whether landmark information will overshadow, or restrict, learning about shape information. Whilst a number of studies have shown that landmarks are not able to overshadow learning about shape information, some have shown that landmarks can, in fact, overshadow learning about shape information. Given the continued importance of theories that grant the shape information that is provided by the boundary of an environment a special status during learning, the experiments presented here were designed to assess whether the relative salience of shape and landmark information could account for the discrepant results of overshadowing studies. In Experiment 1, participants were first trained that either the landmarks within an arena (landmark-relevant), or the shape information provided by the boundary walls of an arena (shape-relevant), were relevant to finding a hidden goal. In a subsequent stage, when novel landmark and shape information were made relevant to finding the hidden goal, landmarks dominated behaviour for those given landmark-relevant training, whereas shape information dominated behaviour for those given shape-relevant training. Experiment 2, which was conducted without prior relevance training, revealed that the landmark cues, unconditionally, dominated behaviour in our task. The results of the present experiments, and the conflicting results from previous overshadowing experiments, are explained in terms of associative models that incorporate an attention variant.

The ability to learn to find a hidden goal on the basis of spatial information is a skill evident in both human and nonhuman animals. For humans, our ability to travel, perhaps many miles, from our homes to our places of work, demonstrates our daily reliance on spatial navigational. For nonhuman animals, the ability to navigate to a source of water or food is necessary for survival. Numerous cues have been shown to aid navigation through an environment, such as internally derived vestibular (e.g., Wallace, Hines, Pellis, & Whishaw, 2002) and somesthetic information (Lavenex & Lavenex, 2010), the slope of the floor (Nardi & Bingman, 2009; Nardi, Newcombe, & Shipley, 2011; Nardi, Nitsch, & Bingman, 2010), landmarks that are both distal and proximal to a goal location (Prados, Redhead, & Pearce, 1999; Roberts & Pearce, 1998; Save & Poucet, 2000), and the shape, or boundaries, of an environment (e.g., Pearce, Ward-Robinson, Good, Fussell, & Aydin, 2001). Landmarks are typically conceived of as discrete objects within an environment, such as a distinctive tree or building, whereas boundary cues, such as a cliff face or the shape created by a walled enclosure, are distinct from landmarks as they tend to confine movement within a particular space.

It is has been shown in a number of experiments that navigation with reference to landmarks follows the principles proposed by associative learning theories. For instance, a landmark close to a goal will restrict what is learned about a landmark that is further away from that goal (Chamizo, Manteiga, Rodrigo, & Mackintosh, 2006; see also Chamizo, Aznar-Casanova, & Artigas, 2003; Gould-Beierle & Kamil, 1999; Leising, Garlick, & Blaisdell, 2011; Roberts & Pearce, 1999; Sanchez-Moreno, Rodrigo, Chamizo, & Mackintosh, 1999; Stahlman & Blaisdell, 2009). The ability of one spatial cue to restrict, or overshadow, what is learned about another spatial cue has led some (e.g., Pearce, 2009) to suggest that learning to navigate is underpinned by the same general associative mechanism as that for nonspatial learning, such as that demonstrated in a variety of classical conditioning experiments (e.g., Jones & Haselgrove, 2011; Pavlov, 1927).

One long-standing, and particularly pervasive, contradiction to this notion, however, is the finding that information provided by the boundary walls of an environment appears immune to overshadowing effects from landmarks. For instance, Doeller and Burgess (2008) conducted an experiment in which participants were required to collect a number of objects within a virtual environment and, having collected the objects, were asked to replace a given object. Distance errors between where the object was replaced and its original position provided a measure of performance. Participants in a compound group were trained in a circular arena that was orientated by distal cues and that contained an intramaze landmark. Following 16 acquisition trials, participants in the compound group were given one of two test phases: for one half of the participants, the circular boundary was removed such that the objects had to be replaced by reference to just the landmark cue, whereas for the other half of the participants, the landmark cue was removed, such that the objects had to be replaced with reference to just the circular boundary. Performance was compared to two control groups that performed the whole experiment with only the landmark, or the circular boundary, as well as the orientation cues. While participants in the compound group who were tested with the circular boundary showed equivalent performance to the boundary control group, participants in the compound group who were tested with the landmark cue displayed greater error than the landmark control group. As such, the circular boundary cue was said to have overshadowed learning about the intramaze landmark, but learning about the circular boundary was immune to overshadowing effects from the intramaze landmarks. Similar effects have been demonstrated in other experiments conducted with humans (Redhead & Hamilton, 2007) and have frequently been demonstrated in experiments conducted with rats (Cheng, 1986; Graham, Good, McGregor, & Pearce, 2006; Hayward, Good, & Pearce, 2004; Hayward, McGregor, Good, & Pearce, 2003; McGregor, Horne, Esber, & Pearce, 2009; Pearce et al., 2001; Wall, Botly, Black, & Shettleworth, 2004) as well as pigeons (Kelly, Spetch, & Heth, 1998).

The apparent inability of landmark cues to interfere with learning about information provided by the boundary walls of an environment has led a number of authors to conclude that boundary information holds a special status when learning to navigate (for reviews see Cheng, 2008; Jeffery, 2010; Lew, 2011; Pearce, 2009). According to both Cheng (1986) and Gallistel (1990), the shape properties of an environment, which are necessarily created by its walls, are processed in a dedicated geometric module that is impervious to the influence of other cues (see also: Wang & Spelke, 2002, 2003). Moreover, learning about the shape of an environment occurs even in situations when other cues are readily available and relevant to finding a goal location. The notion that information provided by the boundary walls of an environment is learned about even in the presence of other predictive cues has recently been echoed by Doeller and Burgess (2008), who state that learning about landmarks follows standard associative principles, but, in contrast, learning about boundaries occurs incidentally and in a manner inconsistent with theories of associative learning (see also: Barry et al., 2006; Burgess, 2006, 2008; Cheng & Newcombe, 2005; Wang & Spelke, 2002; White & McDonald, 2002).

There are, however, a number of problems with using the observation that a landmark is unable to overshadow learning about information provided by boundary walls to conclude that boundary information holds a special, impervious, status when learning to navigate. One objection to such a conclusion is that a failure to observe overshadowing may be accounted for with a mechanism that is, in fact, incorporated into associative theories of learning: generalization decrement (e.g., Pearce, 1987). Consider the compound group in the experiment conducted by Doeller and Burgess (2008), in which the small landmark cue was removed for one half of participants during the test trials. This, potentially, minor change from the conditions of training would lead to the training and test environments appearing visually similar, and, as such, performance may not deteriorate relative to the control group trained with only the boundary wall. In contrast, for the other half of the participants in the compound group, the large circular boundary was removed at test. This more substantial change from the conditions of training could be expected to lead to the training and testing environments appearing visually different. As such, there would be a large deterioration in performance in these participants that would give the impression of an overshadowing effect relative to the control group only trained with a landmark cue. Recent empirical evidence provides a second objection to observing that a landmark is unable to overshadow learning about information provided by boundary walls and then concluding that boundary information holds a special status during navigation. In order to support this contention, there must never be any difference in learning about the boundary of an arena between a compound group, trained with both landmarks and boundary information relevant to the task, and a control group trained that only the boundary walls of an environment are relevant to the task. Contrary to this, there are now a number of published demonstrations of a landmark cue successfully overshadowing learning about shape information provided by the boundaries of an arena (Cole, Gibson, Pollack, & Yates, 2011; Horne, Iordanova, & Pearce, 2010; Horne & Pearce, 2011; Prados, 2011). For example, in an experiment by Pearce, Graham, Good, Jones, and McGregor (2006), an overshadowing group of rats was trained to find a goal that was hidden in one corner of a rectangular arena consisting of two long black walls and two short white walls. Relying on the geometry or the wall colours of each corner would lead the rats to the correct or the geometrically equivalent corner of the rectangle. For a control group, the colour of the short and long walls changed randomly between trials; thus, only geometric information would permit navigation to the correct, or geometrically equivalent, corner. In a test trial conducted in an all-white rectangle, the control group spent significantly longer than the overshadowing group searching in the correct or geometrically equivalent corners.

Any theory that states that information provided by the boundary walls of an environment is learned about independently from landmark cues (e.g., Cheng, 1986; Gallistel, 1990), or in a manner inconsistent with theories of associative theories (e.g., Doeller & Burgess, 2008), struggles to explain instances where landmarks have successfully overshadowed learning about information provided by the boundaries of an environment. There is, however, a need to address why overshadowing experiments conducted within the spatial domain, which have essentially followed the same protocol, produce contradictory findings—especially given that modular theories of geometric and boundary information processing continue to be a matter of theoretical influence (e.g., Doeller & Burgess, 2008; Gallistel & Matzel, 2013; Jeffery, 2010; Spelke & Lee, 2012). In studies of nonspatial learning, the relative salience of two cues presented in compound has been shown to impact upon which cue will take control of behaviour. For example, Mackintosh (1976) trained rats that a compound of a light and a noise signalled an impending shock and compared learning to control groups trained with either the light or the noise in isolation. Throughout the experiment, the intensity of the light was kept constant, but the intensity of the noise was manipulated. In the compound group, a noise of 85 dB overshadowed learning to the light when compared to learning in the light control group. In contrast, the light overshadowed learning about 60 dB or 50 dB noises compared to leaning in noise control groups trained with 60 dB or 50 dB noises, respectively (see also Miles & Jenkins, 1973).

The impact of the relative salience of landmark and boundary cues in determining which cue takes control of behaviour has largely been ignored in the spatial learning literature. We are aware of only one other study that has directly examined the relative salience of landmark and boundary cues in cue competition experiments, which we discuss in the General Discussion. This omission is relatively surprising given the theoretical (e.g., Mackintosh, 1975), and empirical (Mackintosh, 1976; Miles & Jenkins, 1973) relevance that cue salience has on overshadowing. One reason for this oversight, perhaps, is the difficulty in manipulating the unconditional salience of landmark and boundary cues. Whilst it is intuitive to assume that louder noises are more salient than quieter noises, and, thus, when presented in compound with a light to expect that there will be a level of noise intensity at which learning to the light will be overshadowed, it is not clear how to manipulate the unconditional salience of landmark or geometry cues in a similar manner. It might be expected that increasing the size of a landmark would increase its salience, but it is possible to imagine a landmark so large that it would not be an effective cue by which to localize a goal location. Manipulating the wall length ratio of, say, a kite-shaped environment might be a way in which to alter the unconditional salience of a particular corner, but it is possible to imagine a situation where the obtuse corner is almost imperceptible. Even if there were reliable ways of manipulating the salience of landmarks and boundaries, it is not practical, on a participant by participant basis, to judge the relative salience of the two cues a priori and, thus, predict which cue may take control of behaviour. Considering this, it is not unreasonable to suggest that overshadowing experiments conducted within the spatial field are potentially confounded by the issue of relative salience of boundary and landmark cues. If experimenters used boundary cues that were relatively more salient than landmark cues, then it is likely that the landmarks would have failed to overshadow learning about the boundary, a result that, at face value, would be consistent with modular processing of boundary wall information. If, however, experimenters used boundary cues that were relatively less salient than landmark cues, it is likely that the landmarks would have successfully overshadowed learning about boundaries, a result more consistent with an associative analysis of spatial navigation.

The experiments reported here were designed to examine whether the relative salience of landmark and boundary cues could account for why, in some circumstances, landmarks fail to overshadow learning about the boundary walls of an environment and, in other circumstances, successfully overshadow learning about boundary walls. Given the foregoing discussion relating to the difficulty in manipulating the relative unconditional salience of landmark and boundary shape information, salience was manipulated more centrally by driving attention towards a particular cue dimension prior to compound training using a learned-predictiveness procedure. Recent studies conducted in the spatial domain with human (Buckley, Smith, & Haselgrove, 2014) and nonhuman (Cuell, Good, Dopson, Pearce, & Horne, 2012) animals have shown that establishing one spatial cue as predictive of a hidden goal location, and another cue as irrelevant, facilitates subsequent learning about the predictive cue in a manner that is consistent with attentional analyses of learning (e.g., Esber & Haselgrove, 2011; Le Pelley, 2004; Mackintosh, 1975). In Experiment 1, we sought to exploit these observations in order to investigate whether establishing either the landmarks or the geometric properties of the boundary walls as relevant to navigation would influence the relative dominance of these cues when they were subsequently established as equally predictive of a hidden goal during subsequent compound training.

Given that we have noted that the data from previous overshadowing experiments conducted in the spatial domain might be explained via generalization decrement, we did not attempt to assess cue salience through a traditional overshadowing design. Instead, at test, we presented both landmark and boundary information, but placed the two sources of information into spatial conflict with each other (see Method section, Experiment 1). Unlike the overshadowing experiments discussed earlier, as both landmark and boundary cues are presented during the conflict tests, any preference that we observe towards one particular cue domain cannot be explained via generalization decrement. Assessing cue salience via conflict tests also has the additional benefit of being particularly sensitive. When landmark and shape cues, that were previously trained in compound, are presented in isolation, it is possible that participants will search by each cue for a similar amount of time as there is simply no other behaviour to perform during the test. When both cues are presented during the same test, however, participants are given the opportunity to search near both cues. Any slight difference in salience between the cues, which may not be detected when presenting each cue in isolation, would be expected to translate into a preference for searching near one cue over another during a conflict test.

## Experiment 1

In Stage 1 of Experiment 1, participants were required to find a hidden goal that was located in one of the corners of a virtual kite-shaped arena that contained a differently shaded blue sphere in each corner. On every trial, these four spheres changed position. For a landmark-relevant group, the hidden goal was located by the same sphere on each trial during Stage 1; thus, to find the goal participants would have to approach the same landmark regardless of which corner that landmark was in. For a shape-relevant group, the hidden goal was located in the same corner of the kite during each trial of Stage 1. As such, to find the goal participants would have to approach the same corner regardless of which landmark was present at that corner. Experiments conducted in our laboratory (Buckley et al., 2014) have confirmed that this training alters the salience of the landmarks and boundaries of the arena in a manner consistent with attentional models of learning (e.g., Esber & Haselgrove, 2011; Mackintosh, 1975). Thus, for the landmark-relevant group, landmarks will be more salient than the arena boundaries, and vice versa for the shape-relevant group. Following training in the kite, both groups proceeded to Stage 2, during which participants were trained to find a hidden goal in a trapezium-shaped arena that contained a differently shaded red landmark in each corner. The landmarks remained in the same corner throughout each trial; thus, in order to find the hidden goal, participants could rely on: (a) information provided by the landmarks within the arena, (b) information provided by the shape of the arena itself, or (c) a combination of both the landmark and shape cues. To establish which cue dimension, if any, was dominating behaviour, three test trials were intermixed within Stage 2 training trials. During each test trial, in which the hidden goal was not present, the landmark and shape cues were placed in conflict with each other by rotating the configuration of landmarks relative to the boundary shape.

For participants given landmark-relevant training in Stage 1, we expected the landmark cues to be relatively more salient than the shape information provided by the boundary walls at the onset of Stage 2 training. The landmark cue should, therefore, be the more dominant cue during compound training, and, thus, participants would search for longer near the appropriate landmark cue during the conflict test than near the appropriate corner of the shape. In contrast, for those given shape-relevant training in Stage 1, the shape information provided by the boundary walls should be relatively more salient than the landmark cues at the onset of Stage 2 training. The shape information provided by the boundary walls should, therefore, be the more dominant cue during compound training, and, thus, participants should search for longer near the appropriate corner of the arena during the conflict tests than near the landmark cue.

### Method

#### Participants

A total of 24 participants were recruited from the University of Nottingham (18 female). Participants were allocated randomly to either the shape-relevant or landmark-relevant group, with the constraint that the genders were balanced between the two groups. Participants were given course credit or £5 in return for participation. The age of participants ranged from 18 to 27 years (mean = 20.83, *SD* = 2.60). An additional £10 was awarded to the participant who completed Stage 2 of the experiment in the shortest time.

#### Materials

All virtual environments were constructed, compiled, and displayed using Mazesuite software (Ayaz, 2014; Ayaz, Allen, Platek, & Onaral, 2008), and were run on a standard Stone desktop computer, running Microsoft Windows 7. A large Mitsubishi LDT422 V LCD screen (935×527 mm) was used to display the virtual environments. The virtual arenas were viewed from a first-person perspective, all of which had a grass texture applied to the floor. Using the 0–255 RGB scale employed by Mazesuite, the 2.5 m tall cream coloured walls applied to the Stage 1 and Stage 2 arenas were defined as 204, 178, 127. Assuming a walking speed similar to that in the real world (2 m/s), the perimeter of the kite-shaped arena was 72 m, with the small walls being 9 m in length and the long walls 27 m. The kite was configured such that it contained two right-angle corners with the remaining two angles being 143.14° and 36.86°. The perimeter of the isosceles trapezium was 63 m, with the smallest wall being 9 m, the largest wall 27 m, and the remaining two walls 13.5 m in length. The walls were configured such that the isosceles trapezium contained angles of 48.19° and 131.81°.

Four distinctly coloured blue spheres acted as landmarks within the kite-shaped arena, whilst four distinctly coloured red spheres acted as landmarks within the trapezium-shaped arena. All landmarks were 90 cm in diameter and were located 1.48 m away from the apex of each corner, on a notional line that bisected the corner in half. In a horizontal plane, the full 360 degrees of the landmarks were visible during navigation. The spheres were created using Blender software (Blender Foundation, n.d.), and imported into Mazesuite. Using the RGB utilized by Blender, the four blue spheres were defined as RGB 0.000, 0.540, 0.640; 0.159, 0.326, 0.800; 0.000, 0.123, 0.720; and 0.000, 0.464, 0.800, and the four red spheres as RGB 0.635, 0.239, 0.640; 0.640, 0.000, 0.392; 0.512, 0.000, 0.314; and 0.238, 0.131, 0.465. The goals within the arenas were square-shaped regions (1.08 ×1.08 m, invisible to participants), which were also located 1.48 m away from the walls of the arena, along on a notional line that bisected the corner in half. As such, the landmarks were suspended above the hidden goal, and participants were required to walk under the spheres in order to find the hidden goal.

A third arena was also used in this experiment, which was designed to allow participants to become familiar with the controls of the experimental task. This exploration arena was a regular octagon configured with red walls (RGB: 229, 25, 51), with a grass texture again applied to the floor. There was no hidden goal present. Again assuming a walking speed of 2 m/s, each wall was of the exploration arena was 12 m in length.

#### Procedure

After signing a standard consent form, participants were given the following set of instructions on paper:

*This study is assessing human navigation using a computer generated virtual environment. During this experiment, you will complete 43 trials. In each trial, you will be placed into a room that contains an invisible column. Your aim is to end the trials as quickly as possible by walking into the column*.

*You will view the environment from a first person perspective, and be able to walk into the column from any direction using the cursor keys on the keyboard. Once you've found the column a congratulatory message will be displayed and you should hit enter when you're ready to begin the next trial. You will always be in the centre of the arena when a trial begins, but the direction in which you face at the start of each trial will change*.

*To start with, you may find the column is difficult to find. There is, however, a way of learning exactly where the invisible column will be on each trial. It's a good idea to fully explore the environment on the first few trials; this will help you to learn where the column is going to be.*

*This session should take around 15 minutes. If at any point you wish to stop this session, please notify the experimenter and you'll be free to leave without having to give a reason why. Your results will be saved under an anonymous code, and kept confidential throughout*.

*The person who takes the least time to complete this experiment will win a £10 prize!*

Participants sat not more than 100 cm from the screen and were first provided with the opportunity to move around the octagonal exploration arena for two 30-s trials using the four keyboard cursor keys. Presses on the “up” and “down” cursor keys permitted the participant to move forwards and backwards within the arena, respectively. Presses on the “left” and “right” cursor keys permitted the participant to rotate counterclockwise and clockwise within the arena, respectively.

Following these practice trials, participants pressed “enter” to begin the 24 trials of Stage 1 training. On each trial, participants were required to find the hidden goal by using the four cursor keys as described above. There was no time limit on any trial; thus, each trial ended only when the hidden goal was found. Once the hidden goal had been found, participants could no longer move within the arena, and a congratulatory message (*Congratulations, you found the goal!*) was displayed on screen. Participants pressed enter to begin the next trial. In the kite-shaped arena, participants began each trial at a point located halfway between the apex and obtuse corners, and the direction in which participants began facing was randomized for every trial. Generating every possible configuration of four landmarks in the four corners of the arenas produced 24 different trials. Each of these arenas was presented once to each participant, the order of which was randomized for each participant independently. During Stage 1, for participants in the shape-relevant group, the goal was located in the same corner of the kite-shaped arena on each trial. The location of the hidden goal was counterbalanced across participants within this group, such that each corner of the kite signalled the goal location for three participants during the experiment. Each of the four blue spheres was located in the goal corner on six trials and in nongoal locations on the remaining 18 trials. During Stage 1 for participants in the landmark-relevant group, the goal was located under the same blue sphere on each trial. The location of the hidden goal was, again, counterbalanced across participants within this group, such that each of the blue spheres signalled the goal location for three participants during the experiment. Each of the four corners contained the goal on six trials and did not contain the goal on the remaining 18 trials.

**Figure 1.**
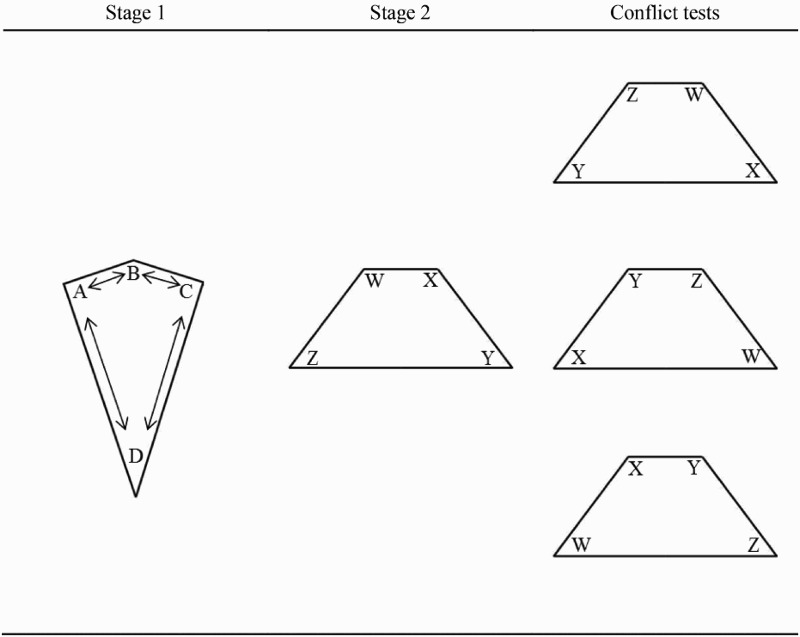
Schematic diagrams of the arenas used in Experiment 1. A, B, C, and D represent the blue spheres that were present within the kite-shaped arena during Stage 1, and the arrows between then represent the fact that the landmarks moved between each of the 24 trials of Stage 1 training. For the landmark-relevant group, the hidden goal remained by a particular sphere, regardless of which corner that sphere was in. For the shape-relevant group, the hidden goal remained in the same corner of the kite, regardless of which sphere was in that corner. W, X, Y, and Z represent the red spheres that were present within the trapezium-shaped arena. The red spheres remained in a constant position during training, such that for every participant, both the corner of the trapezium and the landmark located at that corner signalled the goal location. Finally, during the three test trials, the configuration of red spheres was rotated to place shape and landmark information into conflict.

Having completed 24 training trials in the kite-shaped arena, participants completed Stage 2 of the experiment in a trapezium-shaped arena. Stage 2 consisted of 16 training trials and three conflict test trials. In both training and test, participants began at a point halfway along a notional line from the centre of the shortest wall to the centre of the longest wall; the direction in which participants began facing was randomized on each trial. Figure 1 shows the position of the four red landmarks in the corners of the trapezium arena during training trials. As with Stage 1 training, the location of the hidden goal was counterbalanced across participants within each group. As such, for both the shape- and landmark-relevant groups, the hidden goal was located in each corner of the trapezium for three participants during the experiment. As the red spheres did not move during Stage 2 training, this also meant that each red sphere signalled the goal location for three participants during the experiment. Three 60-second test trials, in which the hidden goal was removed from the arena, were administered after the 8th, 12th, and 16th training trial. On each test trial, the shape and landmark cues were placed into conflict, achieved by rotating the configuration of the four red landmarks relative to the boundary, such that each landmark occupied a different corner to that from training. Rotating the configuration of landmarks by one, two, or three corners in a clockwise direction produced three test trials for each participant. The order of these test trials was counterbalanced across participants such that the one-corner, two-corner, and three-corner rotations were administered equally often during the first, second, or third test trial during the experiment. After 60 s of the test trial had elapsed, participants received a message (*Press enter to start the next trial*), and the next training trial began. Square search zones, three times the area of the hidden goal, were used to measure time spent searching near the correct landmark or near the correct corner during each test trial, a common measure of performance in both animal (e.g., McGregor et al., 2009) and human (e.g., Redhead & Hamilton, 2009) spatial navigation experiments.

### Results

#### Stage 1

**Figure 2.**
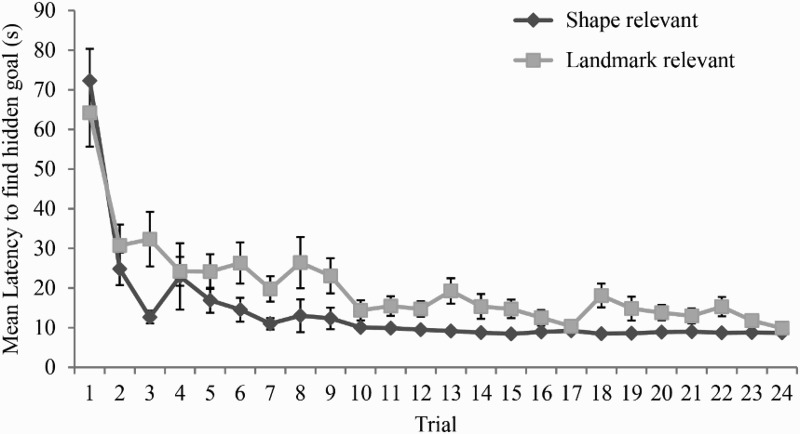
Mean latencies of the two groups to find the hidden goal in Stage 1 of Experiment 1. Error bars show 1 ± standard error of the mean.

Figure 2 shows that mean latency to find the goal, in seconds, for participants in the shape-relevant and landmark-relevant groups during Stage 1 of Experiment 1. In both groups, the latency to find the goal decreased across the 24 training trials in the kite, and there was also an indication that the shape-relevant group found the goal quicker in the kite than did the landmark-relevant group. A two-way analysis of variance (ANOVA) of individual latencies, with the variables of group (landmark-relevant or shape-relevant) and trial (1–24) confirmed these impressions, revealing a significant main effect of trial, *F*(23, 506) = 26.71, *MSE* = 129.31, *p* < .001, ${\text{η}}_{\text{p}}^{2}$ = .55, and group, *F*(1, 22) = 13.90, *MSE* = 399.79, *p* < .005, ${\text{η}}_{\text{p}}^{2}$ = .39. There was no interaction between trial and group, *F*(23, 506) = 1.27, *MSE* = 129.31, *p* = .18.

#### Stage 2

**Figure 3.**
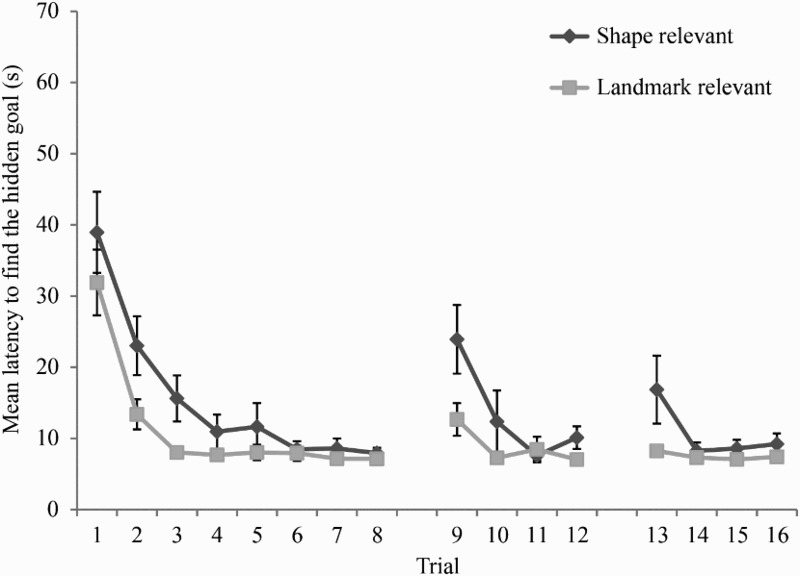
Mean latencies of the two groups to find the hidden goal in Stage 2 of Experiment 1. Error bars show 1 ± standard error of the mean.

Figure 3 shows participants' mean latency to find the goal in Stage 2. Again, the latency to find the goal decreased across the 16 training trials in the trapezium. In the trapezium, there was an indication that the landmark-relevant group found the goal quicker across the course of the experiment than did the shape-relevant group. These impressions were again confirmed by a two-way ANOVA conducted on individual latencies to find the goal, with the variables of group (landmark-relevant or shape-relevant) and trial (1–16), which revealed a significant main effects of trial, *F*(15, 330) = 19.86, *MSE* = 62.10, *p* < .001, ${\text{η}}_{\text{p}}^{2}$ = .47, and group, *F*(1, 22) = 6.87, *MSE* = 232.86, *p* < .05, ${\text{η}}_{\text{p}}^{2}$ = .24, but no interaction between these variables, *F*(15, 330) = 1.31, *MSE* = 62.10, *p* = .20.

#### Test trials

**Figure 4.**
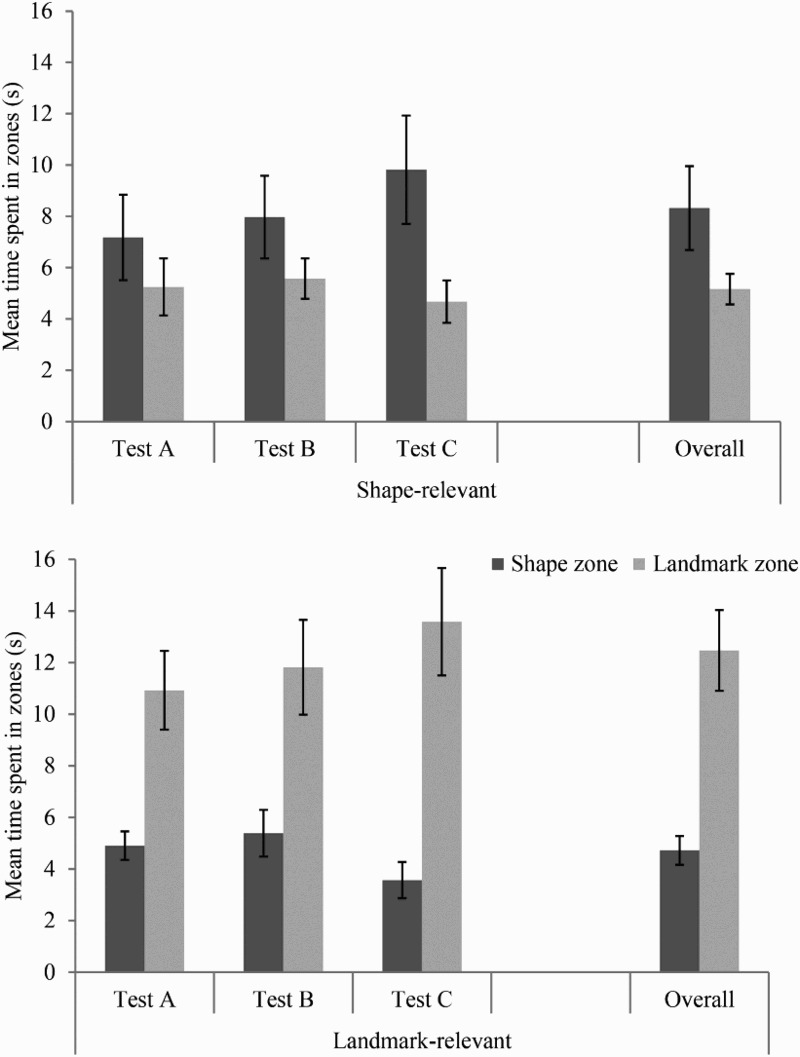
Mean time spent in zones for each of the three conflict tests of Experiment 1for the shape- and landmark-relevant groups. Error bars represent 1± standard error of the mean.

Figure 4 displays the time spent searching in the landmark and shape zones during the three tests by participants in the shape-relevant and landmark-relevant groups. Participants in the shape-relevant group spent more time in the shape than the landmark zone during all three tests. The opposite pattern of results was observed for the landmark-relevant group; here, participants spent longer searching in the landmark than the shape zone during the three tests. In both groups, the bias for searching in one zone over another became stronger in later tests. A three-way ANOVA of individual time spent in zones, with variables of group (shape-relevant or landmark-relevant), zone (shape or landmark), and test (first, second, or third) revealed no significant main effects of group, *F*(1, 22) = 2.32, *MSE* = 40.66, *p* = .14, zone *F*(1, 22) = 2.67, *MSE* = 63.29, *p* = .12, or test *F*(2, 44) = 1.33, *MSE* = 6.97, *p* = .28. There was no significant interaction between test and group, or between test and zone, *F*s < 1. There was, however, a significant interaction between group and zone, *F*(1, 22) = 16.18, *MSE* = 63.29, *p* < .005, ${\text{η}}_{\text{p}}^{2}$ = .11, as well as a significant three-way interaction between group, zone, and test, *F*(2, 44) = 3.51, *MSE* = 13.18, *p* < .05, ${\text{η}}_{\text{p}}^{2}$ = .14. The simple main effects of the three-way interaction that are crucial to our hypotheses regard the time spent in the landmark and shape zones *within* the shape-relevant and landmark-relevant groups, and for the sake of brevity we do not report significant *between-*group effects here. Taking the shape-relevant group first, participants did not show a significant preference for searching in the shape zone over the landmark zone during the first, *F* < 1, or second, *F*(1, 22) = 1.25, *p* = .28, test trials; however, the shape-relevant group did display a preference for searching in the shape zone over the landmark zone during the third test, *F*(1, 22) = 5.09, *p* < .05, ${\text{η}}_{\text{p}}^{2}$ = .19*.* For the landmark-relevant group, participants displayed a significant preference for searching in the landmark zone over the shape zone on each test trial, smallest *F*(1, 22) = 7.08, *p* < .05, ${\text{η}}_{\text{p}}^{2}$ = .24.

### Discussion

Experiment 1 showed that, by establishing a particular cue domain as relevant to navigation, it is possible to bias which cue dimension will dominate subsequent search behaviour. During the conflict tests administered during Stage 2, participants who had received landmark-relevant training in Stage 1 of the experiment searched near the appropriate landmark more than they did near the appropriate corner of the trapezium. In contrast, during the same conflict tests, participants who were given shape-relevant training in Stage 1 of the experiment searched near the appropriate corner more than they did near the appropriate landmark. Importantly, these biases emerged despite both the shape of the arena and the landmarks within it being equally relevant as cues for the location of the hidden goal during Stage 2. Furthermore, as conflict tests were used to assess the relative dominance of the competing cues, in which all the cues employed during training were still presented to participants at test, it is difficult to explain these data by appealing to generalization decrement in its simplest form.

It was evident that the predictiveness training administered in Stage 1 of the experiment produced a stronger effect in the landmark-relevant group than it did in the shape-relevant group. One reason for this might be that the landmark cues in the trapezium were, unconditionally, more salient than the shape properties provided by the boundary walls. If this was the case, landmark-relevance training during Stage 1 of the current experiment would only serve to enhance a preexisting difference in salience. For the shape-relevant group, the training given in Stage 1 should ensure that the attention paid to the shape properties of the boundary walls was higher than the attention paid to the landmark cues at the onset of Stage 2. This manipulation, however, may have been somewhat counteracted by the fact that the landmark cue was, unconditionally, much more salient than the shape information provided by the boundary walls of the trapezium. It is difficult to evaluate this possibility without having a measure of baseline performance, something that we sought to gain from Experiment 2.

## Experiment 2

Participants in the no pretraining group received training identical to that administered in Stage 2 of Experiment 1. As such, participants could rely on either the shape information provided by the boundary walls of the trapezium or the landmarks within it to locate a hidden goal. Again, three conflict tests were administered, in which the landmark cues were placed into conflict with the shape information provided by the boundary walls. If the landmark cues within the trapezium are more salient than the shape information provided by the boundary walls, then participants should spend more time searching near the landmark that had previously signalled the goal location than searching in the corner of the trapezium that had signalled the goal location. In contrast, if the shape information provided by the boundary walls is more salient than the landmark cues, participants should spend more time searching near the corner of the trapezium that had signalled the goal location than near the landmark. Finally, if both cue dimensions are of equal salience, then participants would be expected to spend equal amounts of time searching by the corner of the trapezium that had signalled the goal location and by the landmark that had signalled the goal location.

In addition, we also included a pretraining group who received identical training within the trapezium environment; however, this was preceded by training in a kite-shaped arena. In contrast to Experiment 1, both the shape properties provided by the boundary walls and the landmarks contained with the arena were established as equally relevant for finding the goal. This was achieved by keeping the relationship between the spherical landmarks, the arena corners, and the hidden goal constant on each trial. By including this group, we are able to better match the training in Stage 1 with the two groups of Experiment 1, thus ensuring that participants enter Stage 2 having had experience of navigating in the kite-shaped environment. Attentional theories of associative learning differ in their prediction of the effect of compound training on the salience of the individual cues. According to Mackintosh (1975, see also Esber & Haselgrove, 2011), such training will amplify any unconditional difference in salience between the cues. This follows because attention to a cue will increase if it is the best available predictor of the outcome (in this case the hidden goal) and decrease if it is not. Early on in training, the more salient cue in a compound will enter into an association with the hidden goal quicker than the less salient cue. Consequently, the more salient cue will gain more attention, and the baseline, unconditional, difference in salience between the cues will increase. In contrast, Pearce and Hall (1980) predict that the effect of compound training will be to, at best, sustain any unconditional difference in salience between the cues and, at worst, attenuate their difference. This follows because Pearce and Hall proposed that attention to *each* cue in the compound is equal to the (absolute) total prediction error from the previous trial. As this prediction error will diminish as training progresses, so too will attention to each cue, until they reach an equivalent, low level. In any case, however, these theories do not predict that the effect of compound training will be to reverse any differences in the unconditional salience of cues trained in compound, and, on this basis, the pretraining group should permit an uncompromised measure of cue salience.

As well as allowing us to obtain a measure of baseline performance on our navigation task, which is necessary in order to accurately interpret the data obtained from Experiment 1, Experiment 2 was also theoretically motivated. Previous studies have established that when boundary and landmark information are established as equally predictive of a goal and then subsequently placed in conflict, the boundary cues control navigational behaviour. Cheng (1986) trained rats to find food that was buried in a particular corner of a rectangular arena, the corners of which contained a unique landmark. In one version of his task, Cheng moved the previously relevant landmark to an incorrect geometric corner—placing the boundary shape and landmark cues into conflict. Under these circumstances, rats chose to search in the location signalled by the previously relevant geometry, rather than the location signalled by the previously relevant landmark. Similar findings have also been noted in human adults tested in a real-world circular environment that was orientated by two extramaze cues and that contained an intramaze landmark (Bullens et al., 2010). These findings are consistent with theories that propose that information provided by the boundary shape of an environment should control navigational behaviour, even in the presence of equally relevant cues (e.g., Gallistel, 1990). When viewed in the context of this empirical and theoretical precedent, therefore, it would be particularly surprising if the landmark cues in our task unconditionally control navigational behaviour, at the expense of boundary cues.

### Method

#### Participants

A total of 24 participants were recruited from the University of Nottingham (18 female). Participants were allocated randomly to either the no pretraining or pretraining groups, with the restriction that an equal number of male and females were distributed between the two groups and were matched to the groups of Experiment 1. Participants were again given course credit or £5 in return for participation. The age of participants ranged from 18 to 40 years (mean = 20.88, *SD* = 4.86). An additional £10 was awarded to the participant who completed Stage 2 of the experiment in the shortest time.

#### Materials

All material details were the same as those described for Experiment 1.

#### Procedure

All procedural details, including details pertaining to the exploration arena, were the same as those described in Experiment 1. The no pretraining group received training and conflict tests that were identical to those described for Stage 2 of Experiment 1. The pretraining group also received these trials, but were first required to complete 24 trials in a kite-shaped arena that contained the same four blue landmarks as those detailed in Experiment 1. During these 24 trials, the location of the hidden goal was signalled by both the shape properties provided by the boundary walls of the arena and the landmarks contained within the arena. For all participants in the pretraining group, the hidden goal was located in the right-angled corner of the kite where the left-hand wall was shorter than the right-hand wall. The landmarks within the arena remained static for each participant; however, the location of the landmarks was counterbalanced across participants, such that each blue landmark (A, B, C, and D—see Figure 1) signalled the goal location for three different participants during the experiment.

### Results

Acquisition data from the no pretraining group are analysed together with Stage 2 acquisition data from the pretraining group.

#### Stage 1

The mean latency, in seconds, for participants in the pretraining group to find the goal during Stage 1 of the experiment decreased across the 24 training trials in the kite. A one-way ANOVA of individual latencies, with the variable of trial (1–24), confirmed this impression, *F*(23, 253) = 40.13, *MSE* = 37.38, *p* < .001, ${\text{η}}_{\text{p}}^{2}$ = .79.

#### Stage 2

**Figure 5.**
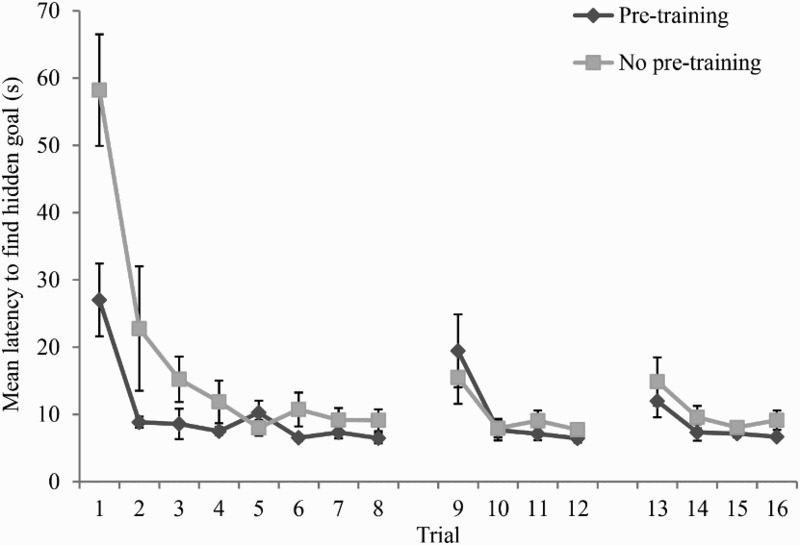
Mean latencies of the two groups to find the hidden goal in Stage 2 of Experiment 2. Error bars show 1 ± standard error of the mean.

Figure 5 shows the mean latency, for participants in both the no pretraining and pretraining groups, to find the goal in Stage 2 of the experiment. Again, the latency to find the goal decreased across the 16 training trials in the trapezium. It was also evident that the pretraining group found the goal quicker than the no pretraining group on early trials. A two-way ANOVA conducted on individual latencies to find the goal, with the variables of group (no pretraining or pretraining) and trial (1–16), revealed significant main effects of trial, *F*(15, 330) = 18.14, *MSE* = 101.02, *p* < .001, ${\text{η}}_{\text{p}}^{2}$ = .45, and group, *F*(1, 22) = 5.62, *MSE* = 334.35, *p* < .05, ${\text{η}}_{\text{p}}^{2}$ = .20, and a significant interaction between trial and group, *F*(15, 330) = 3.93, *MSE* = 101.02, *p* < .001, ${\text{η}}_{\text{p}}^{2}$ = .15. Simple main effects analysis revealed that the pretraining group were quicker to find the goal on Trial 1 only, *F*(1, 22) = 9.93, *MSE* = 588.31, *p* < .01, ${\text{η}}_{\text{p}}^{2}$ = .31.

#### Test trials

**Figure 6.**
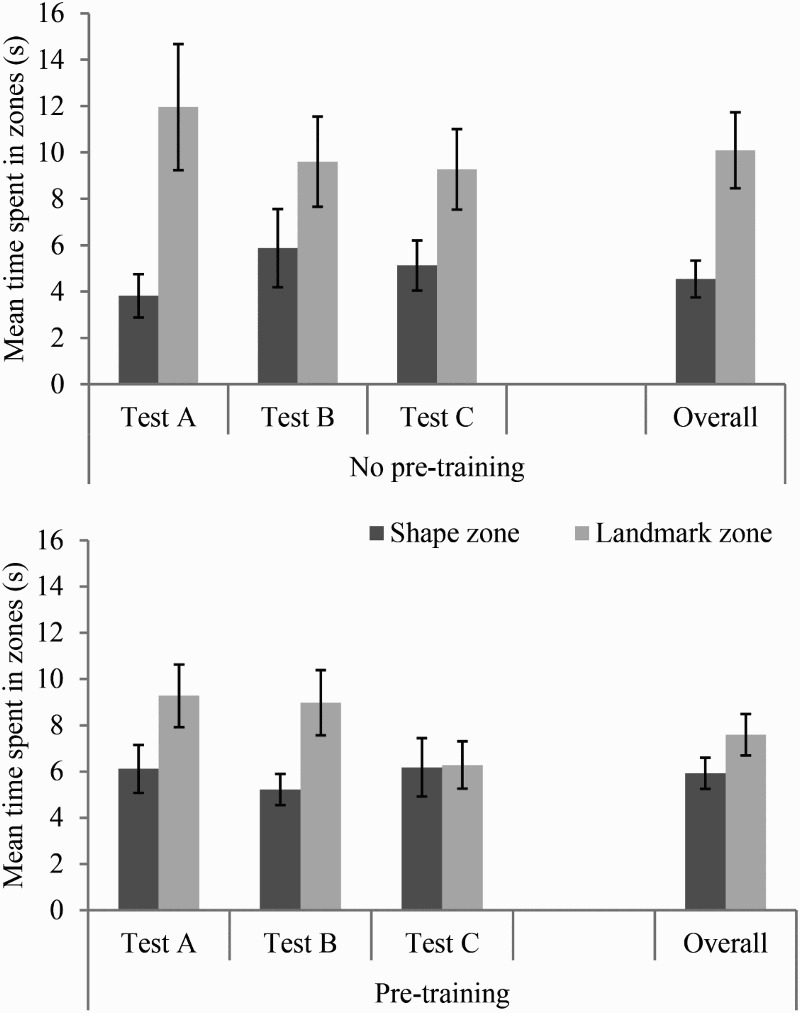
Mean time spent in zones for each of the three conflict tests of Experiment 2 for the no pretraining and pretraining groups. Error bars represent 1± standard error of the mean.

Figure 6 displays, in seconds, the time spent searching in the landmark and shape zones during the three tests of the experiment by participants in the no pretraining and pretraining groups, respectively. Participants in the no pretraining group spent more time searching in the landmark zone, over the shape zone, during the three tests, although this preference for the landmark zone appeared to weaken over the tests. Participants in the pretraining group appeared to initially spend more time searching in the landmark zone over the shape zone. Again, though, this preference weakened over tests and did not appear present during the third test.

Despite these observations, a three-way ANOVA of individual time spent in zones, with variables of group (no pretraining or pretraining), zone (shape or landmark), and test (first, second, or third), revealed only a significant main effect of zone, *F*(1, 22) = 9.81, *MSE* = 54.09, *p* < .01, ${\text{η}}_{\text{p}}^{2}$ = .31, indicating that all participants spent more time searching in the landmark zone than in the shape zone. The main effects of group and test were not significant (both *F*s < 1), nor were the interactions between test and group (*F* < 1), zone and group, *F*(1, 22) = 1.50, *MSE* = 54.09, *p* = .23, or test and zone, *F*(2, 44) = 1.62, *MSE* = 23.16, *p* = .21. Finally, the three-way interaction was not significant, *F* < 1.

### Discussion

During the conflict tests, the no pretraining group of Experiment 2 searched for longer near the landmark cue that previously signalled the goal location than near the corner of the trapezium arena that previously signalled the goal location. As hypothesized, when the shape information provided by the boundary walls of a trapezium arena and the landmarks within the arena are placed into conflict, the landmark cues dominated behaviour—a result we assume to reflect the greater unconditional salience of the landmark cue, relative to the shape information provided by the boundary walls of the environment. A similar pattern of results was also observed in the data obtained from the pretraining group. Again, participants searched for more time near the landmark cue than they did near the correct corner of the trapezium. It appeared that the main effect of zone was carried largely by the no pretraining group. Numerically, at least, the preference for searching near the landmark cue at test was attenuated in the pretraining group, compared to the no pretraining group. This result is consistent with a model of attentional learning that employs a summed error term to determine the attention paid to cues (e.g., Pearce & Hall, 1980); however, we note here the lack of an interaction within our data to substantiate this claim.

That participants favoured searching near the landmark cues, over the boundary cues, contrasts with previous empirical evidence that boundary cues control navigational behaviour in the presence of equally predictive landmark information (e.g., Bullens et al., 2010; Cheng, 1986). Furthermore, it seems difficult to explain these results with theories that suggest that information provided by the boundary shape of an environment should control navigational behaviour, even in the presence of equally relevant cues (e.g., Gallistel, 1990). It may, however, be possible to explain instances where boundary information has dominated navigational behaviour over landmark information, or vice versa, by appealing to associative learning theories that allow for changes in the attention paid to salient stimuli. To avoid undue repetition, we elaborate on this further in the General Discussion.

## General Discussion

Experiment 1 showed that it is possible to manipulate which cue dimension would take control of navigational behaviour in a trapezium-shaped arena that also contained landmarks, by preceding exploration of this environment with relevance training in a different shaped arena, which also contained different landmarks. The shape properties provided by the boundary walls of the environment took control of behaviour if participants had received shape-relevance training prior to learning the goal location in the trapezium. In contrast, the landmark cues within the trapezium took control of behaviour if participants had received landmark-relevance training prior to learning the goal location in the trapezium environment. The effect of relevance training appeared to be asymmetrical, with a greater bias in exploration in the landmark-relevant group. On the basis of this, it was proposed that the unconditional salience of the landmarks was greater than the shape properties provided by the trapezium, and Experiment 2 confirmed this. When learning in the trapezium was preceded by no relevance training altogether, or training in which both shape and landmark cues were relevant, the landmark cues contained within the trapezium took control of behaviour.

The data presented here are inconsistent with theories that suggest that learning about shape information occurs in an impervious geometric module (e.g., Cheng, 1986; Gallistel, 1990), as these theories do not permit learning about landmark information to interact with learning about information provided by boundary walls. Furthermore, the results presented here are also inconsistent with the associative model of spatial navigation proposed by Miller and Shettleworth (2007), as this theory employs a Rescorla and Wagner (1972) learning algorithm (and a choice rule) to determine approach behaviour during spatial navigation. In Experiment 1, an entirely different set of stimuli were used in Stage 2 to those employed during training in Stage 1, and, consequently, any associative strength acquired by the stimuli during training would not directly transfer to the stimuli employed in Stage 2—negating the possibility of their influencing behaviour. Even if generalization of associative strength is permitted between the stimuli used in Stage 1 and Stage 2, this would still not systematically bias search behaviour as the stimuli that were employed as signals for the presence *and absence* of the hidden goal in Stage 1 were established (through appropriate counterbalancing) as equivalently similar to the stimuli that signalled the goal location during Stage 2. Consequently, any propensity for generalization to promote search behaviour near one particular stimulus would be exactly balanced by its propensity to inhibit the same behaviour.

The learned predictiveness effects presented here are, however, consistent with associative models that allow for changes in the attention paid to relevant and irrelevant stimuli, such as that proposed by Mackintosh (1975). According to Mackintosh, cues that are the best predictors of an outcome will enjoy an increase in their salience, or attention, whereas cues that are poor predictors of an outcome will suffer a reduction in their attention (see also: Esber & Haselgrove, 2011; Le Pelley, 2004). Importantly, Mackintosh also proposed that attention generalizes among stimuli in proportion to their similarity (p. 292). Consequently, attention should generalize more between cues that are drawn from the same dimensions than between cues that are drawn from different dimensions. On the basis of this, it is possible to understand the results from Experiment 1. As participants’ navigational behaviour was unconditionally biased towards using the landmark cues in Stage 2 (Experiment 2), administering landmark-relevance training in Stage 1 served to further increase, through generalization, the salience of landmarks contained within the trapezium further, as well as decrease the salience of shape information provided by the boundary walls of the trapezium. This unconditional bias in salience was, seemingly, overcome by the Stage 1 training in the shape-relevant group. For these participants, Mackintosh's theory predicts that the initially salient landmarks will suffer a loss in attention as they are established as irrelevant to navigating towards the goal, and attention to the predictive shape cues will increase. If sufficient training is given, this training should overcome any unconditional biases in salience and, again through generalization, transfer to the cues employed in Stage 2—permitting the establishment of a bias towards learning about the shape of the arena.

The current results provide a proof of concept to the idea that the differing results of spatial overshadowing experiments can be accounted for by the relative salience of landmark and boundary wall cues. Following Mackintosh (1975), it is possible that failures of a landmark to overshadow a boundary shape (e.g., Doeller & Burgess, 2008) and instances in which boundary information has dominated behaviour over landmark information (e.g., Bullens et al., 2010; Cheng, 1986) may be due to the landmark possessing low unconditional salience relative to the shape. Likewise, successes of landmarks overshadowing boundary shape (e.g., Pearce et al., 2006) and instances where landmark cues have dominated navigational behaviour over boundary cues, as observed in Experiment 2, may be due to the landmark possessing high unconditional salience compared to the shape of the arena. One further possibility raised by attentional theories of learning is that failures of landmarks to overshadow learning about information provided by boundary walls may not be limited to instances of salience asymmetry. Mackintosh (1976) noted that, if both cues enter an experiment with particularly high unconditional salience, then they will be limited in their ability to undergo a further increase in attention. This will have the consequence of permitting them to acquire an equivalent association with the trial outcome as a cue that is trained in isolation, thus limiting the degree to which overshadowing can be observed. Consequently, if both the landmark and shape cues in previous overshadowing experiments were both of an unconditionally high salience, then the landmark would fail to overshadow learning based upon the shape of the boundary, and vice versa. Evidence consistent with this general prediction about the influence of stimulus salience on overshadowing was obtained in a nonspatial learning experiment reported by Mackintosh (1976), who demonstrated that overshadowing of conditioned suppression in rats was obtained between two stimuli when they were both of a low unconditioned salience but not when they were both of a high unconditional salience. It remains to be determined whether a comparable effect can be observed in the spatial domain.

It is relevant, at this point, to discuss our results in relation to empirical data gathered from other spatial learning experiments. Our findings are consistent with overshadowing studies in which a landmark has successfully overshadowed learning about the shape properties provided by the boundary walls of an environment (Cole et al., 2011; Horne et al., 2010; Horne & Pearce, 2011; Pearce et al., 2006; Prados, 2011). We observed a similar effect in Experiment 2, where landmarks dominated behaviour over the shape properties provided by the boundary walls of the arena. However, we observed this by comparing performance in a direct manner, via a series of conflict tests, rather than via a traditional overshadowing design, in which navigation using only the boundary walls of the environment is compared, following either landmark–boundary wall compound training, or training with just the boundary walls alone. These conflict tests permit us to obtain a measure of which cue has taken control over behaviour when the confounding effects of generalization decrement are less apparent. Of more theoretical importance, the results gathered here complement experimental data gathered from rats (Kosaki, Austen, & McGregor, 2013) and extend the findings to human participants. Kosaki et al. (2013) elegantly demonstrated that the obtuse corners of a rhombus were less salient than the acute corners, before demonstrating that discrete landmarks were able to overshadow the less salient obtuse corner, but not the more salient acute corner. Taken together, our results and those of Kosaki et al. suggest that spatial cues of superior salience take control of navigational behaviour in a manner that is partially consistent with the predictions made by associative theories of navigation (e.g., Miller & Shettleworth, 2007). Importantly, though, the current results are consistent with other experiments, both in humans (Buckley et al., 2014) and in rats (Cuell et al., 2012), which suggest that associative models of spatial navigation need to acknowledge the role of a more top-down attentional process in learning. That is, associative models must permit changes in the attention paid to a stimulus to be driven both by the inherent properties of that stimulus, in a bottom-up manner envisaged by the Miller–Shettleworth model, and also by more central changes in attention that are a consequence of learning about that stimulus, as proposed by attentional models (e.g., Mackintosh, 1975).

It is important to note that the current results were obtained with landmarks that were discrete from the boundary walls. In the rat literature particularly, coloured wall panels have been conceived as landmark cues. It has, however, been claimed that it might be better to conceive of coloured walls panels as an aspect of boundary information (Wilson & Alexander, 2010) and, moreover, that nongeometric cues that also provide information about the geometry of an arena may be incorporated into a representation of the overall shape of an environment (Cheng & Newcombe, 2005). Consequently, it would be possible to claim that experiments that have studied the interaction between the geometric properties afforded by boundary walls, and the colour of those walls, might have been assessing cue competition within a boundary wall module. As the landmark cues in our experiment were not integrated into the boundary structure, but were instead discrete objects contained within the boundary walls of the arenas, it is difficult to argue that our landmarks could be processed within such a boundary wall module. Consequently, our data show clear cue competition between landmark and geometric cues in a manner inconsistent with theories that suggest that boundary and landmark cues are processed separately (e.g., Cheng, 1986; Gallistel, 1990; Wang & Spelke, 2002, 2003).

To conclude, the experiments reported here, together with overshadowing experiments such as those reported by Kosaki et al. (2013), suggest that the same associative processes as those that explain learning in the nonspatial literature may also explain spatial learning phenomena. Associative theories are able to explain successful observations of cue competition effects between shape information provided by boundary walls and landmark cues, an experimental phenomenon that is inconsistent with theories that state that information provided by the boundary walls of an environment is learned about independently from landmark cues (e.g., Cheng, 1986; Gallistel, 1990), or those that state this information is learned about in a manner inconsistent with associative learning theories (e.g., Doeller & Burgess, 2008). More importantly, considering the continued importance of modular theories (e.g., Gallistel & Matzel, 2013; Jeffery, 2010; Spelke & Lee, 2012), the development of associative accounts of spatial navigation that incorporate an attentional variant will provide the necessary framework to explain the absence of overshadowing between landmarks and shape information without recourse to specialized processing of certain cues.
